# Cohort profile: the German Diabetes Study (GDS)

**DOI:** 10.1186/s12933-016-0374-9

**Published:** 2016-04-07

**Authors:** Julia Szendroedi, Aaruni Saxena, Katharina S. Weber, Klaus Strassburger, Christian Herder, Volker Burkart, Bettina Nowotny, Andrea Icks, Oliver Kuss, Dan Ziegler, Hadi Al-Hasani, Karsten Müssig, Michael Roden, The GDS Group

**Affiliations:** Institute for Clinical Diabetology, Leibniz Institute for Diabetes Research, German Diabetes Center at Heinrich Heine University, Düsseldorf, Germany; German Center for Diabetes Research (DZD), München-Neuherberg, Germany; Department of Endocrinology and Diabetology, Medical Faculty, Heinrich Heine University, Düsseldorf, Germany; Institute for Biometrics and Epidemiology, Leibniz Institute for Diabetes Research, German Diabetes Center at Heinrich Heine University, Düsseldorf, Germany; Public Health Unit, Medical Faculty, Heinrich Heine University, Düsseldorf, Germany; Institute for Clinical Biochemistry and Pathobiochemistry German Diabetes Center, Leibniz Institute for Diabetes Research, Düsseldorf, Germany

**Keywords:** Insulin resistance, Magnetic resonance spectroscopy, Beta cell function, Metabolic phenotyping, Diabetes comorbidities

## Abstract

**Background:**

The German Diabetes Study (GDS) is a prospective longitudinal cohort study describing the impact of subphenotypes on the course of the disease. GDS aims at identifying prognostic factors and mechanisms underlying the development of related comorbidities.

**Study design and methods:**

The study comprises intensive phenotyping within 12 months after clinical diagnosis, at 5-year intervals for 20 years and annual telephone interviews in between. Dynamic tests, including glucagon, mixed meal, intravenous glucose tolerance and hyperinsulinemic clamp tests, serve to assess beta-cell function and tissue-specific insulin sensitivity. Magnetic resonance imaging and multinuclei spectroscopy allow quantifying whole-body fat distribution, tissue-specific lipid deposition and energy metabolism. Comprehensive analyses of microvascular (nerve, eye, kidney) and macrovascular (endothelial, cardiorespiratory) morphology and function enable identification and monitoring of comorbidities. The GDS biobank stores specimens from blood, stool, skeletal muscle, subcutaneous adipose tissue and skin for future analyses including multiomics, expression profiles and histology. Repeated questionnaires on socioeconomic conditions, patient-reported outcomes as quality of life, health-related behavior as physical activity and nutritional habits are a specific asset of GDS. This study will recruit 3000 patients and a group of humans without familiy history of diabetes. 237 type 1 and 456 type 2 diabetes patients have been already included.

**Electronic supplementary material:**

The online version of this article (doi:10.1186/s12933-016-0374-9) contains supplementary material, which is available to authorized users.

## Background

### Why was the cohort set up?

Diabetes mellitus (DM), particularly type 2 DM, is a global health issue affecting about 387 million people [1]. Also type 1 DM, characterized by insulin deficiency due to autoimmune-mediated beta-cell destruction, is rising [2]. All DM types tightly associate with microvascular (retinopathy, nephropathy, neuropathy) and macrovascular comorbidities (ischemic heart disease, peripheral vascular disease, cerebrovascular disease) and several malignancies. The resulting organ dysfunctions affect at least one-third of individuals with DM accounting for approximately 8 % of global all-cause mortality in humans aged between 20 and 79 years [3].

#### Subphenotypes of DM

Type 2 DM is characterized by a long “prediabetic” state with impaired insulin sensitivity, which promotes hyperinsulinemia. With failing insulin secretion, glycemia increases until diagnostic thresholds for overt diabetes are exceeded [4]. There is evidence for the existence of subphenotypes even in the prediabetic state, possibly resulting from differences in the pathogenesis, and also with regard to the course of disease and related complications. In most forms of DM, beta cell dysfunction is a major driving force for disease development and progression [5]. Despite extensive research on potential underlying mechanisms, such as glucose- or lipid-mediated toxicity [6, 7], mitochondrial dysfunction [8] or inflammation [9] the processes involved in beta cell failure are not fully understood [10]. Defects in pancreatic beta-cell function are often preceded by insulin resistance [11–13] which is not only a feature of type 2 DM, but is also found in patients with type 1 DM [14, 15]. Moreover, obesity associates with increased incidence of type 1 DM [16, 17], which has led to the term ‘double diabetes’. The mechanisms linking type 1 DM and insulin resistance, and thereby type 2 DM, are yet unknown.

Gene variants identified by genome-wide association studies seem to affect DM susceptibility predominantly through beta-cell dysfunction but also with insulin resistance [18]. The risk alleles are common in the population, but their effect size is small and not suitable for general genetic screening [19]. Identification of genetic determinants for insulin resistance relied on its surrogate markers such as fasting insulin [20, 21]. If such genetic variants are studied in comprehensively phenotyped cohorts [22], novel therapy targets might be identified [23]. The distribution of subphenotypes, the predictive value for the development of comorbidities and the effectiveness of stratified or personalized treatment strategies are yet unclear.

#### Complications and diagnosis of early manifestations

In individuals at low risk of cardiovascular diseases, i.e. younger non-smoking normotensive women, the relative cardiovascular risk is multiplied by the occurrence of type 2 DM [24]. Individuals with newly diagnosed type 2 DM already present comorbidities presumably due to longer-term undetected hyperglycemia [25], while some patients with type 1 DM show a rapid progress of diabetes-associated diseases [26] despite good metabolic control [27]. Regarding macrovascular diseases, the prognostic importance of the metabolic syndrome compared to the sum of its individual components has been challenged [28]. Even the obvious link between hyperglycemia and comorbidities appears complex [29]. The predictive value of subphenotypes for the development of comorbidities and the effectiveness of individualized treatment strategies is far from being understood. GDS focusses on the presumably formative early period after diagnosis of the disease and the thorough prospective assessment of (pre) clinical manifestations of comorbidities over 10 years.

#### Individualized intervention strategies

There is evidence for sustained protection by intensive glycemic control early after onset of DM, with reduction of mortality and micro- and macrovascular comorbidities decades thereafter, referred to as legacy effect [24, 29]. Current glucose-lowering drugs have modest efficacy on diabetes endpoints, so novel therapy strategies need to prove additional positive effects on the development of comorbidities.

Nutritional behavior is an important lifestyle factor influencing the risk of developing type 2 DM [30] and to some extent of type 1 DM [31, 32]. Dietary intervention in type 2 DM can improve insulin sensitivity and beta-cell function [33]. Accordingly, excess availability of certain metabolites such as free fatty acids and branched-chain amino acids induces whole-body insulin resistance [34–36]. Together with gene variants related to response to lifestyle intervention [37], metabolome profiling will have impact for the development of treatment and prevention strategies. The GDS has the potential to imply targeted interventions in selected subgroups covering a broad range of potential mechanisms as well as diabetic comorbidities.

#### Classical and novel risk factors

Despite the vast amount of data on classical risk factors, the impact of novel biomarkers, e.g. identified by multiomics technologies or innovative imaging tools is incompletely understood [38, 39]. The majority of epidemiological studies have estimated insulin resistance from surrogate parameters, yielding incorrect results in patients with impaired beta-cell function [40–42]. Therefore, we aimed at standardizing methods for comprehensive metabolic phenotyping and implementation of novel tools in the initial period between 2005 and 2009. The updated validated study program started in 2009 with a focus on cellular mechanisms of insulin resistance. High lipid availability and ectopic lipid deposition in skeletal muscle and the liver play a central role in the development of insulin resistance [43] but the cellular mechanisms remain unknown [44, 45]. Regulators of subcellular lipid partitioning and mitochondrial oxidation strongly determine insulin sensitivity but are only partially understood [46].

Both DM and its comorbidities have in common that inflammation-related processes are involved in their development [47–49]. The current knowledge on the relevance of biomarkers of subclinical inflammation is mainly restricted to studies on hard cardiovascular endpoints in patients with type 2 DM [47–50]. Prospective data are limited on associations of inflammation and quantitative phenotypes in earlier stages of cardiovascular disease and all stages of microvascular comorbidities or cognitive decline and are mainly derived from cohorts of patients with type 1 DM. We hypothesize that circulating biomarkers of subclinical inflammation predict the deterioration of insulin sensitivity and beta-cell function and the progression of micro- and macrovascular comorbidities in patients with newly diagnosed DM.

For the identification of novel predictors, most previous studies included patients with longer DM duration [51–53]. Therefore, we acquire circulating biomarkers of subclinical inflammation, non-invasive data on energy and lipid metabolism in liver and skeletal muscle and store biopsy samples of skeletal muscle and subcutaneous adipose tissue for future analyses.

Sociodemographic and psychosocial determinants have been discussed as modifiable regulators of the course of DM. These include e.g. socioeconomic position, health-related quality of life and mental disorders [54–57]. Knowledge about the changes of innovative patient reported measures such as patients’ preferences, information needs and time needed for health-related activities [58, 59] during the progression of the disease is lacking so far.

The aims for setting up the GDS were identification of (i) sub-phenotypes of DM with respect to insulin sensitivity, insulin secretion and (ii) predictors of early diabetes-related comorbidities, (iii) to develop individualized intervention strategies for the treatment and prevention of diabetes and related comorbidities (iv) to analyze the impact of known and novel risk factors (i.e. nutrition, subclinical inflammation, energy metabolism, body fat distribution, metabolites and other biomarkers, socioeconomic and psychosocial conditions) on disease progression and development of diabetes-related comorbidities, in recently diagnosed patients with type 1 and type 2 DM. GDS will test the hypothesis that the course of DM and related comorbidities is determined by processes that depend on diabetes type, affecting immunological factors, energy homeostasis, body fat composition and distribution and patient reported measures.

## Study design and methods

The GDS is an ongoing prospective observational study comprising intensive phenotyping within 12 months after clinical diagnosis, at 5-year intervals for at least 20 years and annual telephone interviews in between. The study is performed according to the Declaration of Helsinki, approved by the ethics committee of the University of Düsseldorf (previous reference number 2478, current reference number 4508) and was registered at Clinicaltrials.gov (Identifier number: NCT01055093).

### Who is in the cohort?

The primary inclusion criterion is diagnosis of DM according to current ADA recommendations [60] within the last 12 months in individuals aged between 18 and 69 years. These include maturity onset diabetes of the young (MODY) and latent autoimmune diabetes of the adult (LADA), while individuals suffering from type 3 (e.g. pancreoprive DM) or type 4 (gestational) DM are not included. The main inclusion criteria are provided in detail in Table 1. The recruitment was performed via advertisements in local newspapers and the institutional homepage and via practitioners we supplied with information and flyers. After receiving the contact details, the potential participants were contacted and prescreened in a detailed telephone interview to check the inclusion and exclusion criteria. Appropriate applicants were then invited to the first study day including physical examination and anamnesis, thereafter participation in the more advanced examination was decided upon. From 2015 on a glucose tolerant subgroup matched for sex, BMI and age will be included to the data base. Participants give written informed consent to the study protocol.

**Table 1:** Key exclusion criteria

Key inclusion criteria	Key exclusion criteria	Exclusion criteria for specific examinations
Diagnosis of type 1 DM and type 2 DM including maturity onset diabetes of the young (MODY) and latent autoimmune diabetes of the adult (LADA) based on current ADA recommendations^a^ Onset of DM within the last 12 months Diagnosis of type 1 DM based on diabetes manifestation with ketoacidosis or immediate insulin requirement along with the presence of at least one islet cell directed autoantibody or C-peptide levels below detection limit^a^ Age of 18–69 years	Secondary DM according to ADA criteria (Type 3 B-H, e.g. pancreoprive DM) Type 4 (gestational) DM, pregnancy Poor glycemic control (HbA1c > 9.0 %) Hyperlipidemia (triglycerides and low-density lipoproteins ≥double upper reference limit) Heart, renal, liver failure (NYHA ≥II, serum creatinine ≥1.6 mg/dl, Aspartate-Aminotransferase/Alanine-Aminotransferase/Gamma-Glutamyltransferase Peripheral artery occlusive disease IV Venous thromboembolic events Anemia, blood donation or participation in a clinical study within the past 3 months Acute infection, leukocytosis, immunosuppressive therapy, autoimmune diseases, infection with human immunodeficiency virus, other severe diseases (e.g. active cancer disease) Psychiatric disorders, limited cooperation ability	Neurologic examination: corneal disorders, and neuropathy from causes other than diabetes Spiroergometry: electrocardiogram abnormalities (alterations of the ST segment, higher-grade arrhythmia), unstable angina pectoris, uncontrolled hypertonia Magnetic resonance spectroscopy/imaging: metallic implants (cardiac pacemaker or defibrillator, cochlear implants, implanted catheters, clips, prosthetic valves), metallic fragments (metal removed from eye, ever worked as metal worker), larger tattoos, waist circumference > 135 cm, claustrophobia Tissue biopsies: effective anticoagulation therapy, platelet aggregation inhibitors >100 mg acetylsalicylate

^a^ American Diabetes Association [60]

By 01/2015 240 type 1 and 458 type 2 DM patients and three patients with MODY were included. GDS (in Germany named Deutsche Diabetes-Studie, DDS) was initiated at the German Diabetes Center (Deutsches Diabetes Zentrum, DDZ), Leibniz Center for Diabetes Research at Heinrich Heine University, Düsseldorf, Germany and developed into a national multicenter study with ongoing inclusion of patients from different regions of Germany being a core research project within the German Center of Diabetes Research (DZD e.V.) since 2014. The other partners and associates of DZD e.V. now contributing to GDS with increasing numbers include alphabetically listed the Department of Endocrinology/University Hospital Schleswig–Holstein; Department of Medicine I and Clinical Chemistry, University of Heidelberg; Faculty of Medicine/University Hospital Carl Gustav Carus, Dresden; Faculty of Medicine/Ludwig Maximilians University, Munich; German Institute of Human Nutrition Potsdam Rehbruecke; and Institute for Diabetes Research and Metabolic Diseases at Eberhard Karls University, Tübingen.

### How often will they be followed up?

All patients undergo the full test program at baseline, at 5 and 10 years after diagnosis, and shall be continued thereafter. In between, a standardized telephone interview is conducted in annual intervals. During the initial phase of the study, the full test program was also repeated after 2 years in 137 patients. The annual telephone interviews reach a response rate of around 83 %. The 5-year follow-up started in 2014 and shows a response rate of 61 %. Another 33 % of patients are potential responders as they are still within the allowed time frame, while 6 % are lost to follow-up. Of those lost to follow up, 48 % refrained from further participation, 33 % were not accessible, 11 % died, 4 % refused for lack of time and 4 % showed non-compliance to the protocol.

### What is measured?

In addition to demographic data (Table 2), baseline and follow up assessments include clinical and metabolic variables at baseline and follow up, summarized in Table 3. To address the issue of beta-cell dysfunction, the phenotyping includes assessment of beta-cell function using the glucagon stimulation test and mixed meal tolerance test (MMTT) [61]. In addition, the Botnia clamp consists of an intravenous glucose tolerance test (IVGTT [62]) for further testing of beta-cell function followed by a hyperinsulinemic-euglycemic clamp test providing precise dynamic measures of insulin secretion and sensitivity [63, 64]. Hepatic insulin sensitivity is measured by co-infusion of [6,6-^2^H_2_]glucose [65]. Cycling ergospirometry is performed to measure cardiorespiratory performance [37], indirect calorimetry to assess energy expenditure and substrate oxidation during fasting and hyperinsulinemia [66]. The experimental protocols are described in the Additional file 1: Annexure 1.

**Table 2:** Baseline characteristics of type 1 and type 2 diabetes patients

	Type 1 DM	Type 2 DM
Age	(%)	(%)
15–19	5.4	0.2
20–24	15.0	1.1
25–29	16.7	1.7
30–34	18.0	3.1
35–39	10.0	4.6
40–44	9.6	8.5
45–49	8.7	15.3
50–54	10.4	16.2
55–59	2.5	17.2
60–64	3.0	18.8
65–69	0.8	13.1
70–74	0	0.2
Sex		
Male	62.5	67.0
Female	37.5	33.0
Marital status		
Single	55.0	21.8
Co-habiting/married	40.8	61.6
Separated/divorced	3.7	12.4
Widowed	0.4	4.1
Higher education		
Up to class 8	9.6	27.1
Junior high school	20.0	22.5
Up to class 10	0.0	5.0
Advanced technical college	13.3	12.2
Secondary school examination	55.0	30.6
No education	0.4	0.7
Other types of education	1.2	1.7
No response*	0.4	0.2
Employment		
Laborer	9.2	16.2
Employee	54.6	61.3
Official	5.4	5.0
Business man	7.2	10.0
Farmer	0	0.2
Self-employed worker	2.1	3.5
Non-employed	0.8	0
Other professions	5.4	1.5
No response/advanced education^a^	1.2	0.2
Medical insurance		
Private	15.8	12.4
Government/public	77.9	81.9
Other insurance	5.8	4.6
No insurance	0	0
No response	0.5	1.1
Regular medical checks		
Yes	35.8	56.1
No	63.7	42.8
No response^a^	0.4	0.9
Family history of diabetes		
Mother	16.1	32.7
Father	17.3	26.3
Children	0.8	1.7
Brothers and sisters	7.6	18.4
Grandparents	37.3	35.2
Uncles and aunts	15.8	20.0
Other diseases/risk factors/comorbidities		
Hypertension	18.5	63.3
History of myocardial infarction	0.4	2.8
Retinopathy	0.8	1.3
History of or current smoking	73.3	88.9
Subclinical DSPN	9.8	6.9
Confirmed asymptomatic DSPN	0.4	2.6
Confirmed symptomatic DSPN	2.2	4.0
Possible DSPN	7.2	23.2
Probable DSPN	0.5	5.3
Subclinical/borderline CAN	0.9	2.1
Definite CAN	1.4	2.4
Medication		
Glucose lowering therapy		
Insulin, short acting	87.9	5.9
Insulin, long acting	54.2	4.8
Metformin	14.6	56.1
Sulfonylurea	1.3	3.5
Dipeptidyl-peptidase-4 inhibitors	1.3	6.6
GLP-1-Agonists	0.4	2.0
Other therapies		
Acetylsalicylic acid	1.7	11.1
Statins	2.5	17.5
Fibrates	0.0	0.7
Any antihypertensive therapy		
Blockers of the renin-angiotensin system	2.1	15.9
Beta blockers	2.9	25.3
Calcium channel blockers	0.8	13.8
Diuretics	0.4	8.3

Demographic characteristics of the type 1 and type 2 diabetes mellitus (DM) patients included until 01/2015 at baseline in  % of whole study group. DSPN: diabetic sensorimotor polyneuropathy, CAN: cardiovascular autonomic neuropathy. DSPN and CAN were defined as previously reported (68, 98)

^a^No response refers to the number of participants, who prefer not to answer to a specific question during the interview

**Table 3:** Clinical parameters at baseline in (a) type 1 and (b) 2 diabetes patients

	N	M ± SD	LQ/UQ	Med
*Panel a*				
Age (years)	240	36.0 ± 11.8	26.3/45.4	34.0
Body mass index (kg/m^2^)	240	24.8 ± 4.1	22.0/26.6	24.0
Waist circumference (cm)	239	86.1 ± 12.6	76.2/94.0	85.0
Hemoglobin A1c (%)	237	6.5 ± 1.2	5.8/6.9	6.3
Glucose (mg/dl)	232	133.4 ± 48.0	106.0/150.5	121.0
Total cholesterol (mg/dl)	238	184.9 ± 38.6	160.5/207.5	180.5
HDL cholesterol (mg/dl)	236	60.5 ± 17.3	48.5/70.5	59
LDL cholesterol (mg/dl)	236	108.9 ± 33.5	87.0/126.5	105.0
Triglycerides (mg/dl)	238	89.6 ± 58.2	56.0/102.0	74.0
ASAT (U/l)	238	22.4 ± 8.5	17.0/25.0	20.2
ALAT (U/l)	238	25.2 ± 18.5	15.9/28.0	20.9
GGT (U/l)	238	22.1 ± 21.7	11.1/26.0	16.0
hsCRP (mg/dl)	237	0.2 ± 0.3	0.1/0.2	0.1
C-peptide (ng/ml)	236	1.2 ± 0.9±	0.5/1.4/	1.0
SBP (mmHg)	237	129.5 ± 14.9	120.5/138.0	129.0
DBP (mmHg)	237	78.0 ± 9.8	71.0/84.0	77.0
VO_2_max (ml min.^−1^ kg^−1^)	203	27.0 ± 7.8	22.1/31.3	25.7
FMD (%)	201	6.8 ± 6.6	2.2/10.6	5.6
NMD (%)	196	16.4 ± 9.5	9.3/22.1	15.8
*Panel b*				
Age (years)	458	53.5 ± 10.4	47.1/62.4	54.8
Body mass index (kg/m^2^)	456	31.7 ± 6.0	27.1/35.4	31.1
Waist circumference (cm)	455	105.9 ± 14.7	95.0/115.5	105.5
Hemoglobin A1c (%)	452	6.4 ± 0.8	5.8/6.8	6.2
Fasting glucose (mg/dl)	438	125.0 ± 28.6	107.0/137.0	122.0
Total cholesterol (mg/dl)	451	206.2 ± 42.0	180.0/234.0	203.0
HDL cholesterol (mg/dl)	448	46.4 ± 12.8	37.0/53.0	45.0
LDL cholesterol (mg/dl)	448	130.5 ± 36.1	105.2/153.5	129.0
Triglycerides (mg/dl)	451	176.0 ± 165.2	98.0/203.8	137.0
ASAT (U/l)	451	25.4 ± 11.0	19.0/29.0	23.0
ALAT (U/l)	451	34.5 ± 19.5	21.9/41.8	29.0
GGT (U/l)	451	43.0 ± 51.0	21.4/48.0	31.6
hsCRP (mg/dl)	446	0.4 ± 0.7	0.1/0.5	0.3
C-peptide (ng/ml)	445	3.3 ± 1.6	2.2/4.2	3.0
SBP (mmHg)	447	141.6 ± 17.1	129.5/152.0	141.5
DBP (mmHg)	447	85.1 ± 10.5	78.0/91.5	84.5
VO_2_max (ml min.^−1^ kg^−1^)	317	19.1 ± 4.9	15.6/21.8	18.7
FMD (%)	332	5.6 ± 5.3	1.9/8.25	4.5
NMD (%)	338	12.2 ± 7.3	6.6/16.2	11.5

Number (N) of type 1 (DM) participants or samples, *M* mean, *SD* standard deviation, *Med* median, *LQ* lower quartile, *UQ* upper quartile, *HDL* high-density lipoprotein, *LDL* low-density lipoprotein, *ASAT* Aspartate-Aminotransferase, *ALAT* Alanine-Aminotransferase, *GGT* Gamma-Glutamyltransferase, *hsCRP* high sensitive C-reactive protein, *SBP* systolic blood pressure, *DBP* diastolic blood pressure. *VO*_*2*_ *max* maximal oxygen uptake, *FMD* flow-mediated (endothelium-dependent) vasodilatation, *NMD* nitrogen-mediated (endothelium independent) vasodilatation

RNA and DNA samples are purified from whole-blood and peripheral blood mononuclear cells (PBMCs), serum and plasma samples (citrate, EDTA) are stored at −80 °C for analysis of biomarkers [67, 68]. Neurological and cutaneous microvascular assessments include nerve conduction studies, quantitative sensory testing, neuropathic symptoms and deficits, heart rate variability, baroreflex sensitivity, pupillography, sudomotor function, sexual function, intraepidermal nerve fiber density, corneal confocal microscopy, laser Doppler flowmetry, skin autofluorescence [69–72], opthalmological examinations include funduscopy, corneal esthesiometry [73] and optical coherence tomography (OCT) [74].

In mid-2012 acquisition of skeletal muscle and subcutaneous abdominal adipose tissue samples and magnetic resonance imaging and spectroscopy (MRI/S) examinations were implemented. Absolute quantification of phosphorus metabolites in the liver and recovery of phosphocreatine after depletion through exercise in skeletal muscle have been applied for the estimation of tissue specific energy metabolism [75]. Assessment of whole body fat distribution and ectopic lipid storage is assessed from proton spectroscopy and MRI.

Patient-reported outcomes are assessed via questionnaires at baseline and follow-up investigations and during annual telephone interviews (Table 3), reflecting quantitative analysis of lifestyle, course of the disease, compliance and socio-economic factors associated with the disease, and dietary habits. Participation preferences, information needs and time for health related activities are assessed by the Control Preferences Scale, the Autonomy Preference Index and questionnaires developed and validated in the DDZ, and diabetes self-management instruments which are well established in cohort studies [76–80]. Quality of life and depression are assessed by common questionnaires (Table 4).

**Table 4:** Questionnaires at baseline and follow-up

	Baseline	5-year follow-up	10-year follow-up	Telephone interview
Demographics				
Age	Y	N	N	N
Sex	Y	N	N	N
Marital status	Y	Y	Y	Y
Retardation/physical disabilities	Y	Y	Y	Y
Diabetes				
Time of diagnosis	Y	N	N	N
Symptoms at time of diagnosis	Y	N	N	N
Diabetes treatment regime	Y	Y	Y	Y
Diet plan and advice	Y	Y	Y	Y
Diabetes education for the patients	Y	Y	Y	Y
Ophthalmological complications	Y	Y	Y	Y
Kidney complications	Y	Y	Y	Y
Cardiovascular complications	Y	Y	Y	Y
Neurological complications	Y	Y	Y	Y
Cerebrovascular complications	Y	Y	Y	Y
Radiation exposure in last 10 years	Y	Y	Y	Y
Family history of diabetes and other diseases	Y	Y	Y	Y
Socio-economic status				
Household composition	Y	Y	Y	Y
Education	Y	Y	Y	Y
Health insurance	Y	Y	Y	Y
Employment	Y	Y	Y	Y
Net household income	Y	Y	Y	Y
Personal health behavior, life style				
Smoking	Y	Y	Y	Y
Alcohol	Y	Y	Y	Y
Physical activity	Y	Y	Y	Y
Food frequency questionnaire	Y	Y	Y	Y
Regular medical checks	Y	Y	Y	Y
Personal health history				
Other diseases	Y	Y	Y	Y
Food supplement intake	Y	Y	Y	Y
Other medication	Y	Y	Y	Y
Self reported weight and weight change	Y	Y	Y	Y
Mental health	Y	Y	Y	Y
Reproductive history	Y	Y	Y	Y
Health-related quality of life				
WHO-5, SF-36	Y	Y	Y	Y
WHOQUOL-Bref, SCL-14	Y	Y	Y	N
Depression				
PHQ, PAID, ADS-L	Y	Y	Y	Y
Information needs, patient time				
CPS, API				

Questions and questionnaires at baseline, 5-year follow-up, 10-year follow-up, and annual telephone interview. *Y* stands for ‘yes’ (question asked) and *N* stands for ‘no’ (question not asked)

Short form 36 health survey questionnaire (SF36), World Health Organization 5 questions health survey (WHO5), World Health Organisation quality of life assessment (WHOQOL-Bref), symptom checklist 14 (SCL-14), patient health questionnaire (PHQ), problem areas in diabetes (PAID), Allgemeine Depressionsskala (ADS-L), Control Preferences Scale (CPS), Autonomy Preference Index (API)

## Discussion

### What has it found? key findings and publications

#### Baseline characteristics

The basal characteristics of the cohort recruited between 01/2009 and 1/2015 are summarized in Table 2. In our cohort, the percentage of patients diagnosed with type 2 DM at an age of less than 45 years, commonly referred to as “early manifestation of type 2 DM” is doubled compared to US registry data (Table 2) [81–83]. This might result from the higher willingness of younger persons to participate in time-consuming examinations. Such higher motivation could also reflect their higher educational levels compared to other cohorts of newly diagnosed type 2 DM. Accordingly, mean age at diagnosis was comparable to cohort studies that excluded elderly patients [84, 85] (Table 2b). The percentage of male type 2 DM participants is higher compared to other cohorts, which might result from more frequent manifestation in males <65 years [86–88]. Our observational study is not designed as a population-based study and therefore does not claim to represent the German diabetes population, but intends to reveal predictors of later outcome in specific subgroups and to unravel underlying mechanisms. We now post this statement and compare our baseline description of the first included patients to one report of Hartwig et al., who reviewed four large representative regional cohorts of incidental type 2 diabetes and the data from the national German Health Interview and Examination Survey for Adults (DEGS), which combines a nationally representative health survey and a longitudinal follow-up of participants from the previous German National Health Interview and Examination Survey in 1997–1999 [89]. The participants of our cohort include younger patients and more males than represented in this reference study, while the body mass index is comparable. The mean body mass index at baseline is also comparable to other European cohorts [84, 86–88].

**Fig. 1:**
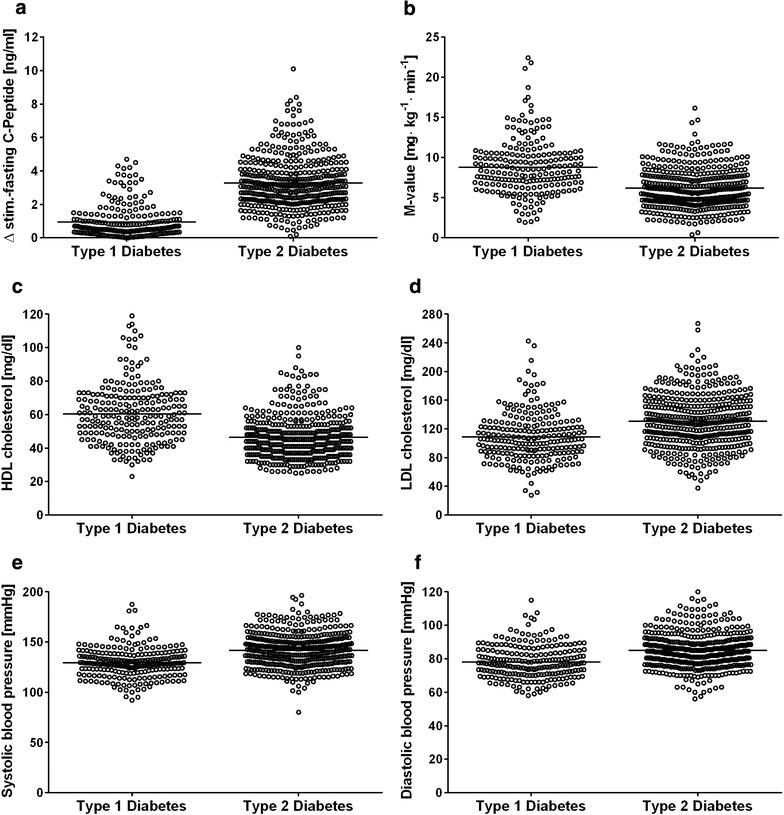
Beta-cell function as assessed from increase of C-peptide during the glucagon stimulation test (C-peptide concentration at 6 min after injection minus C-peptide concentration at baseline) (**a**, n = 216 patients with type 1 DM, n = 374 patients with type 2 DM), insulin sensitivity (M value) as assessed from euglycemic-hyperinsulinemic clamp tests in patients with type 1 DM or type 2 DM (**b**, n = 204 patients with type 1 DM, n = 351 patients with type 2 DM), high-density lipoprotein levels (HDL, **c**, n = 236 patients with type 1 DM, n = 448 patients with type 2 DM), low-density lipoprotein levels (LDL,** d**, n = 236 patients with type 1 DM, n = 448 patients with type 2 DM), systolic blood pressure (**e**, n = 237 patients with type 1 DM, n = 447 patients with type 2 DM), and diastolic blood pressure (**f**, n = 237 patients with type 1 DM, n = 447 patients with type 2 DM)

The mean body mass index at baseline is comparable to other European cohorts [84, 86–88]. A high proportion of type 2 DM patients demonstrated evidence of modifiable cardiovascular risk factors at diagnosis. Participants with newly diagnosed type 1 DM were leaner and younger compared to type 2 participants (Table 2a). In line with type 1 DM intervention studies, a remarkable number still had residual beta-cell function (Fig. 1a) [90–93]. We reported the possible effects of low-grad inflammation and dietary habits on maintenance of residual beta-cell function in follow-up examinations 2 years after diagnosis [94]. Future follow-up examinations will unravel the role of other factors, that are currently under discussion, body fat distribution i.e. liver fat content. On the other hand, we found that a substantial number of type 1 DM patients showed decreased insulin sensitivity, which is in line with previous reports [14, 95] and might relate to poor glycemic control [96] or other mechanisms also attributable to type 2 DM [97] (Fig. 1b). In addition to insulin treatment 15 % of type 1 DM patients were treated with metformin, a few had oral glucose lowering agents and 8 % were not treated with insulin yet (Table 2a). These data show that type 1 DM patients may have oral glucose lowering at diagnosis of the disease before establishing the correct diagnosis. Cardiorespiratory fitness as assessed from oxygen uptake at maximal workload during spiroergometry (VO_2_max) is often impaired in patients with type 2 DM [98]. Accordingly, VO_2_max was lower in patients with type 2 DM compared to patients with type 1 DM and corresponded to values reported previously (Table 3a, b). Reduced VO_2_max might also be due to reduced mitochondrial oxidative capacity, sedentary life style and possibly due to higher hepatocellular lipids and might predispose to the development of insulin resistance [99, 100]. Inverse correlation of VO_2_max with IL-6 and hsCRP in healthy men [101] and with hsCRP, white blood cell count and fibrinogen in men with T2D [102] suggest that lower VO_2_max might predispose to the development of cardiovascular disease in diabetes [103].

Endothelial-mediated (flow-mediated) and endothelial-independent (nitrogen-induced) vasodilatation of patients with type 2 DM (Table 3b) were in the range of previous studies in patients with type 2 DM without previous cardiovascular events [104] and higher compared to baseline parameters in a number of statin intervention studies [105] which might be due to the shorter period of diabetes, the lower cholesterol levels, better glycemic control and the inclusion of patients undergoing statin treatment, factors that are known to positively correlate with vasodilatation. Endothelial function of type 1 DM patients corresponded to values in healthy control of similar body mass and age [104].

Overall in case of type 2 DM patients, 41.9 % had plasma triglycerides >150 mg/dl, 23.3 % had plasma low-density lipoproteins <160 > 150 mg/dl, 73.6 % had blood pressure >130/85 mmHg and 62.5 % were overweight or obese (Fig. 1c–f). At baseline, 18 % of type 2 DM patients were receiving a statin, 9 % were receiving a fibrate, 11 % were being treated with an acetylsalicylic acid agent, and 55 % were receiving any drug for blood pressure reduction (Table 2). The percentage of statin use is low, particularly in type 2 diabetes patients and cardiovascular risk factors are not perfectly controlled at the onset of diabetes (Fig. 1; Table 2).

#### Summary of the results obtained so far

By beginning of 2015, GDS yielded 13 original articles published in peer-reviewed journals [35, 64, 66, 69–71, 75, 106–111].

#### Standardization of experimental protocols

- The measures of insulin sensitivity derived from the Botnia clamp were validated against the standard hyperinsulinemic-euglycemic clamps in patients with type 2 DM [64].
- Post-calorimetric individual calibration procedures have been developed to increase the accuracy and comparability of indirect calorimetry assessed in different centers [66].
- Blood glucose measuring instruments were validated against gold-standard method and the method with the highest accuracy was selected [107].

#### Establishment of novel methods for metabolic imaging

- At DDZ, noninvasive phosphorous (^31^P) MRS of liver was established and optimized with short examination time on a 3-T clinical magnet [75]. With this method, GDS started to employ quantifying absolute concentrations of hepatic adenosine triphosphate (ATP) and inorganic phosphate (Pi) as measures of liver energy metabolism [112].
- Rapid methods for the quantification of hepatic and pancreatic fat were developed applicable to larger cohorts, showing that no relationship exists between pancreatic adipose tissue infiltration and beta cell function, regardless of glucose tolerance status [109, 110].

#### Development of comorbidities

- Immune cell phenotyping showed distinct occurrence of certain white blood cell subtypes and associations with insulin sensitivity, glycemia and lipidemia in patients with type 1 and type 2 DM [106].
- The sensitivity of an indicator test for sudomotor dysfunction on the foot (Neuropad) for detecting small fiber dysfunction was relatively high in recently diagnosed type 1 DM (80 %) and somewhat lower in type 2 DM (68 %) (64). Thus, early sudomotor dysfunction may be demonstrated by screening in recent-onset diabetes.
- Using novel methods to quantify small nerve fiber density (NFD) including corneal confocal microscopy and skin biopsy early nerve pathology was detected in up to 20 % of subgroups with type 2 DM participating in the GDS [69–71]. However, the vast majority of patients with abnormal corneal NFD showed concomitantly normal intraepidermal NFD and vice versa. Thus, both techniques detect early nerve fiber loss in recently diagnosed type 2 DM, but largely in different patients, suggesting a patchy manifestation pattern of small fiber neuropathy. Recently diagnosed type 2 DM patients also demonstrate a marked reduction of cutaneous Langerhans cell density, which relates to insulin resistance in women [69]. Prospective data will establish whether the initial Langerhans cell decline could promote a cutaneous immunogenic imbalance toward inflammation predisposing to polyneuropathy and foot ulcers. Moreover, dermal expression of mitochondrial superoxide dismutase (SOD2) expression in the lower limbs was augmented by ≈60 % and correlated with increasing diabetes duration, cardiac sympathetic predominance, and diminished vagal activity, while subepidermal endothelial cell area was not altered. The SOD2 overexpression points to an early enhanced, presumably compensatory, cutaneous anti-oxidative defence in type 2 DM [111]. Whether cutaneous SOD2 levels can predict the development of diabetic neuropathy will be determined during the prospective GDS follow-up.
- Assessing various single nucleotide polymorphisms (SNPs) in the transketolase gene, we observed associations of genetic variability in transketolase enzyme with neuropathic symptoms and reduced thermal sensation in the GDS baseline cohort, suggesting a role of pathways metabolizing glycolytic intermediates in early diabetic neuropathy.
- Using the diagnostic criteria for diabetic sensorimotor polyneuropathy (DSPN) based on the Toronto Consensus (85), the prevalence of DSPN was relatively high, achieving 20 % in individuals with type 1 DM and 42 % in those with type 2 DM (Table 3). DSPN was subclinical in 10 % of the type 1 DM subjects and possible in 23 % of those with type 2 DM. The prevalence of confirmed DSPN was relatively low, with 3 % in individuals with type 1 DM and 7 % in those with type 2 DM, similar to the prevalence of cardiovascular autonomic neuropathy (CAN) at 2 % in type 1 DM and 5 % in type 2 DM patients. The rate of DSPN strongly depends on the definition of DSPN and is considerably lower, if both clinical and electrodiagnostic criteria are combined. The prevalence of definite CAN in GDS participants with type 2 DM (2.4 %) is similar to the rate of 1.8 % observed by the Verona Newly Diagnosed Type 2 Diabetes Study (VNDS) [113]. Likewise, the prevalence of DSPN found in the present study is compatible with the percentages of 4–39 % depending on the different definition criteria for DSPN used in cohorts of newly diagnosed DM patients [114].
- Biomarkers of subclinical inflammation are associated with DSPN and both motor and sensory nerve conduction velocity (NCV). High serum IL-6 was associated with the presence of DSPN and reduced motor NCV in type 2 DM. In addition, higher levels of high-molecular weight (HMW) and total adiponectin were consistently associated with DSPN and both reduced motor and sensory NCV in individuals with type 2 DM. In participants with type 1 DM however, associations between high adiponectin and higher motor NCV were found. Thus, our data support the hypothesis that the pathomechanisms leading to DSPN may only partially overlap between type 1 and type 2 DM [115].

#### Cellular mechanisms of insulin resistance

- In a subgroup of type 2 DM patients, we assessed cellular mechanisms of insulin resistance in skeletal muscle [35]. These data provided evidence that specific diacylglycerol species underlie activation of protein kinase C, which impairs insulin signaling.
- In a subgroup of type 2 DM participants, we analysed effect of low-caloric interventions on insulin sensitivity and found that energy restriction per se seems to be key for improving insulin action in phases of active weight loss in obese type 2 DM, with a potential improvement of subclinical inflammation with a diet free of red meat [108].
- Higher levels of biomarkers of subclinical and vascular inflammation were found associated with the deterioration of glycemic control and decreases in beta-cell function in study participants with recently diagnosed type 1 and type 2 DM [94].

### What are the main strengths and weaknesses?

The main strength is the broad spectrum of comprehensive metabolic phenotyping combining gold standard methods with novel techniques in humans shortly after diagnosis of DM at regular intervals for 20 years. The examinations combine highly specialized tests, such as non-invasive cutting-edge metabolic imaging, micro methods applied to tissue biobanks and blood samples, which have not been used in previous long-term studies. The array of morphological and functional measures allows for recording preclinical occurrence of comorbidities and will allow for large scale interventional studies.

Detailed test results are sent to both participants and their physicians, and diabetes information days are organized at DDZ to keep patients informed and to increase compliance of the participants.

One weakness is that GDS includes only people living in Germany and cannot represent all ethnicities (Additional file 2: Annexure 2).

Another challenge is present by potential sample size limitations. Since the GDS study is not a randomized trial with a single pre-specified hypothesis, but a very complex cohort study with many observed and calculated variables, all exemplarily performed sample size calculations were based on a logistic regression model for a binary response and a single binary or continuous covariate. Standard regression analyses in cohort studies in general involve additional covariates for confounder adjustment. Therefore, all initially calculated sample sizes can easily be inflated by a variance inflation factor (VIF) and a factor for the expected dropout rate in the study [116]. As an example, considering as a response the incidence of peripheral diabetic neuropathy (DPN) within 10 years after baseline and a properly/poorly regulated HbA1c value (defined as ≤/> 6.5 %) as covariate, further, assuming an annual incidence of DPN of approx. 2 %, a poorly regulated HbA1c in the GDS cohort of 42.5 %, and an odds ratio of 1.95 to be clinically relevant, a test at level α = 0.05 with power of 80 % to detect this odds ratio if 480 probands are included in the cohort. when additionally allowing for confounder adjustment by a VIF of 20 % and a dropout rate of 40 %, then at least 999 participants are required.

Potential selection bias includes higher social standards, the acquisition of scientific knowledge and gathering more information on the disease being the most important motivation for the participants since the study protocol is time consuming and demanding and includes invasive examinations at low expense allowance. Deep phenotyping results in detailed information that is provided to the practitioner. This may induce a change of the treatment strategy. Baseline assessment, yearly phone interviews and reassessments every 5 years might increase motivation to adhere to the therapy recommendations and thereby might refer to as a kind of intervention compared to other patients that were not included. Thus, in addition to the mentioned selection bias, these factors might confound the outcome measures. The participants received the results on the clinical examinations such as beta cell function, insulin sensitivity, cardiorespiratory fitness, liver fat content, (pre)clinical onset of retinopathy or neuropathy, assessment of dietary habits and laboratory measures and had the opportunity to discuss these findings with a medical doctor and a nutritionist. Assessment of preclinical manifestation of comorbidities may remain without distinct immediate clinical consequence. Incidental findings, e.g. abnormalities found during magnetic resonance imaging are communicated to the participant and the practitioner for further diagnostic steps to be taken. At the same time the investigator is not obligated to assess clinical findings beneath the scope of the research question. This approach is approved by the ethical committee and explained to the participants prior to inclusion.

### Can I get hold of the data? Where can I find out more?

A request and transfer process has been established so that researches may apply for data by contacting the study coordinators via email (GDS@ddz.uni-duesseldorf.de). Once approved by the steering committee, the requesting researcher and the principal investigator of GDS sign a contract on the terms and conditions of data transfer and transmission of results back to the DDZ.

## Additional files

10.1186/s12933-016-0374-9 Methods.
10.1186/s12933-016-0374-9 Risk analysis and risk mitigation measures.
