# Supplementary material for: Cohort profile: the German Diabetes Study (GDS)
Source: Cardiovasc Diabetol. 2016 Apr 7;15:59. doi: 10.1186/s12933-016-0374-9 (PMC4823856; doi:10.1186/s12933-016-0374-9)
Supplement: Supplementary file 1 — 10.1186/s12933-016-0374-9 Methods. [file 12933_2016_374_MOESM1_ESM.docx]

**Annexure I: Methods**

**1. Anthropometric measurements**

Body height and weight is measured using a calibrated scale with stadiometer (SECA674, Hamburg, Germany) without shoes and in light underwear. Measurement is done horizontally with a non-elastic tape with waist circumference taken midway between lower costal arch and the superior anterior iliac spine and hip circumference measured at the widest point over the greater trochanters.

**2. Dynamic metabolic tests**

Metabolic tests are carried out on two separate tests in random order. During dynamic metabolic tests, blood glucose concentrations are measured on-site (EKF biosen C-Line glucose analyzer, EKF diagnostic GmbH, Barleben, Germany) ([1](#_ENREF_1)).

**2.1. Assessment of Beta-cell function**

**2.1.1. Glucagon stimulation test**

This test assesses glucagon-dependent insulin secretion and provides a measure of beta-cell function. Blood samples are taken before (0 min), and 6 min after injecting 1 mg glucagon (GlukaGen, NovoNordisk, Mainz, Germany) within 60 sec into the antecubital vein. Glucagon-stimulated insulin secretion is assessed as the difference between or the ratio of insulin (C-peptide) concentrations at 6 min and 0 min ([2](#_ENREF_2)).

**2.1.2. Mixed meal tolerance test (MMTT)**

The mixed meal tolerance test (MMTT) was shown to be more suitable for assessing beta cell function in type 1 diabetes mellitus (DM) compared to the glucagon stimulation test ([2](#_ENREF_2)). The MMTT uses 6 ml/kg (maximum 360 ml) of Boost High Protein (Nestlé HealthCare Nutrition, Osthofen, Germany) ingested within 5 min. Blood sampling for plasma glucose, insulin, C-peptide, glucagon, incretins, triglycerides, and free fatty acids is done at two time points before and every 30 min until 180 min after meal ingestion.

**2.1.3. Intravenous glucose tolerance test (IVGTT)**

The intravenous glucose tolerance test (IVGTT) allows assessment of the early and late phase of insulin secretion to assess beta cell function ([3](#_ENREF_3)). After baseline blood sampling, a bolus of glucose (1 mg/kg body weight in a 30% solution; Glucosteril, Fresenius Kabi, Bad Homburg, Germany) is injected within 60 sec into the antecubital vein at 0 min and blood samples are obtained at 2-min intervals for the first 10 min and thereafter every 10 min until 60 min. First phase C-peptide secretion is calculated as the incremental area under the curve (iAUC) until 10 min, second phase C-peptide secretion as the iAUC between 10 and 60 min, the total C-peptide secretion as the sum of both.

**2.2. Assessment of Tissue-specific Insulin Sensitivity**

**2.2.1. Hyperinsulinemic-euglycemic clamp test**

The hyperinsulinemic-euglycemic clamp test is the gold-standard measure for whole-body insulin sensitivity ([4](#_ENREF_4)). According to the Botnia-clamp protocol ([5](#_ENREF_5)), the clamp test starts directly after the IVGTT. The priming dose (10 mU*body weight [kg]^-1^*min^-1^ for 10 min) is followed by a constant infusion (1.5 mU*body weight [kg]^-1^*min^-1^ for a minimum of 32 hours) of short-acting human insulin (Insuman^®^ Rapid sanofi-aventis Frankfurt am Main Germany) ([6](#_ENREF_6)). Blood sampling for plasma glucose is done every 5 min and an intravenous infusion of 20% glucose is adjusted to maintain the blood glucose concentration at 5 mmol/l during the clamp test. Whole-body glucose disposal (M-value) is given as space-corrected mean glucose infusion rate during the last 30 min of the clamp.

**2.2.2. Hepatic insulin sensitivity**

Hepatic insulin sensitivity is measured as insulin suppressed endogenous glucose production by using a continuous infusion of deuterated glucose (D-[6,6-^2^H_2_]-glucose) (Cambridge Isotope Lab., Andover, Mass., USA; 99% ^2^H enriched) ([7](#_ENREF_7)). The infusion is started two hours before the IVGTT by a bolus application for 10 min with a subsequently continuous infusion aiming at enrichment of 2%. All of the infusions used thereafter within the IVGTT and the clamp also need to be enriched by 2% of D-[6,6-^2^H_2_]-glucose. Determination of atom percent enrichment (APE) of ^2^H and calculation of endogenous glucose production are done as described before ([8](#_ENREF_8)). The analyses are performed on a Hewlett-Packard 6890 gas chromatograph equipped with a 25-m CPSil5CB capillary column (0.2 mm i. d., 0.12 µm film thickness; Chrompack/Varian, Middelburg, The Netherlands) and interfaced to a Hewlett Packard 5975 mass selective detector.

**3. Clinical chemistry**

All venous blood samples are processed and stored under standardized quality-assured conditions. The whole spectrum of standard parameters of clinical chemistry, including plasma glucose, cholesterol (total, high-density lipoproteins, low-density lipoproteins), serum triglycerides, gamma glutamyl-transferase, aspartate and alanine aminotransferase, amylase, lipase, creatine kinase, cystatin C etc. were measured on a Hitachi 912 analyzer (Roche Diagnostics, Mannheim, Germany) in the beginning of the study and is now measured on a cobas c311 (Roche Diagnostics, Mannheim, Germany). Parameters for coagulation and for differentiation of blood cells are determined on a Sysmex CA500 and XP300, repectively (Siemens Healthcare Diagnostics, Erlangen, Germany). Hemoglobin A1c is measured on a Variant-II (Bio-Rad, Munich, Germany). Targeted metabolic profiling of blood metabolites is performed with the X MetaDis/DQTM Kit at Biocrates Life Sciences (Innsbruck, Austria).

**4. Biobank**

**4.1. Plasma, serum and stool samples**

Serum insulin was initially measured using a chemoluminimetric microparticle enzyme-immunoassay (Immulite1000 XPi, Siemens, Erlangen, Germany), C-peptide by a MEIA assay (Axsym, 182 Abbott, Wiesbaden, Germany), later both of them on a Immulite 2000 XPi. Serum and plasma concentrations of non esterified fatty acids, incretines, inflammation-related biomarkers (C-reactive protein, pro- and anti-inflammatory cytokines, adipokines, soluble adhesion molecules) are analyzed using established enzyme-linked immunosorbent assays (ELISAs), bead-based multiplex assays and assays for automated analyzers ([9](#_ENREF_9), [10](#_ENREF_10)). After collection faecal samples are stored at -20° C until further analysis. DNA extraction is performed by using a BioRobot EZ1 machine (Qiagen, Hilden, Germany) according to the manufacturer’s instructions and stored at -80° C to perform next-generation sequencing for further analysis of gut microbial composition ([11](#_ENREF_11)).

**4.2. Genomics**

Fasting blood samples are collected in PAXgene Blood RNA tubes (PreAnalytiX; Hombrechtikon, Switzerland) for transcriptome-wide gene expression analyses (mRNA, miRNA) using microarray-based and sequencing methods ([12](#_ENREF_12)). DNA samples are acquired from whole blood and peripheral blood mononuclear cells (PBMC).

**4.3. Skeletal muscle biopsy**

The region above the vastus lateralis of the quadriceps muscles is anaesthetized with local anaesthetics (lidocaine 2%) ([13](#_ENREF_13)). Thereafter, a muscle biopsy sample of ~70-400 mg is obtained using a Bergström needle. Samples are immediately stored in liquid nitrogen and then frozen at -80°C until analyses. Thereafter the incision is bandaged.

**4.4. Adipose tissue biopsy**

The subcutaneous abdominal adipose tissue biopsy (about 0.5 – 2 g) is taken after local anesthesia of the periumbilical area using 2% of lidocaine solution. Adipose tissue is sampled through a small skin incision using an aspiration needle. Samples are blotted free of blood and connective tissue and immediately stored in liquid nitrogen and then frozen at -80°C until analyses.

**5. Metabolic imaging**

Magnetic resonance imaging (MRI) and multinuclei spectroscopy (^1^H/^31^P-MRS) are performed on a clinical 3 Tesla (Philips X-series Achieva, Best, The Netherlands) whole body magnet equipped with multinuclear capability at the German Diabetes Center (GDC) Düsseldorf, Germany. In addition a 1.5 Tesla (Magnetom, Siemens Healthcare, Erlangen, Germany) whole body magnet is used to measure body fat distribution, liver fat and intramyocellular lipids at the Institute for Diabetes Research and Metabolic Diseases at Eberhard-Karls-University, Tübingen.

**5.1. Body fat distribution**

Whole body fat distribution is measured using magnetic resonance imaging (MRI). Participants are positioned in the magnet in the prone position. A standard transverse multislice turbo spin echo sequence is used to acquire images with bright fat contrast using the quadrature body coil. Images are manually analyzed using the segmentation tool of the Philips operator console (software release 3.2.1.1) to quantify total subcutaneous and visceral adipose tissue volumes.

**5.2. Ectopic fat**

Liver fat is measured with the stimulated echo acquisition mode (STEAM) proton magnetic resonance spectroscopy (^1^H-MRS) sequence, as previously described ([14](#_ENREF_14)). In short, non-water suppressed spectra are acquired from a 3×3×2 cm^3^ volume of interest with water and fat peaks integrated using the jMRUI v4.0 software. Intramyocellular lipids (IMCL) are measured by ^1^H-MRS from the tibialis anterior muscle and soleus muscle using the point resolved spectroscopy sequence (PRESS) from a localized volume of interest with a repetition time (TR) of 2000 ms and an echo time (TE) of 32 ms, with 16 and 96 signal averages, respectively. Spectra are analyzed using LCmodel to obtain a measure of IMCL in reference to tissue water content.

**6. Assessment of in vivo energy metabolism**

**6.1. Whole body substrate oxidation**

The open circuit indirect calorimetry allows to noninvasively determine total energy expenditure, glucose and lipid oxidation by respiratory gas analysis. Following a resting period of 10 minutes, the canopy of the respiratory gas analyzer (Vmax Encore 29n, Sensor Medics Corp., Homestead, FL, USA) is placed over the volunteers' head to measure the oxygen uptake (VO_2_) and the carbon dioxide output (VCO_2_) in vivo under resting conditions. The procedure is repeated during the last 30 minutes of the hyperinsulinemic-euglycemic clamp to assess metabolic flexibility of substrate oxidation ([15](#_ENREF_15)). To optimize the accuracy of the results, an individual calibration (ICcE) for each volunteer is conducted ([16](#_ENREF_16)). The ICcE-corrected values for the primary parameter VCO_2_ and VO_2_ allow to calculate the corresponding resting energy expenditure (REE) by using the *Weir equation* (REE = (3,941*V_O2_+1.11*V_CO2_)*1.44) ([16](#_ENREF_16)).

**6.2. Skeletal muscle oxidative capacity**

Skeletal muscle phosphocreatine (PCr) recovery in vastus lateralis is measured using phosphorus (^31^P) MRS, as previously described ([17](#_ENREF_17)). In short, a knee-extension protocol is performed on a MR compatible ergometer during acquisition of PCr kinetics. The knee extension is performed for 5 min with a weight corresponding to 50-60% of the subject’s maximal knee-extension capacity.

**6.3. Hepatic phosphorous metabolites**

Liver ATP content is measured using ^31^P-MRS, as previously described ([14](#_ENREF_14)). In short, spectra are acquired using a 14 cm circular ^31^P surface coil (transmit-receive coil, Philips Healthcare, Best, Netherlands), using the ^1^H body coil for ^1^H-decoupling and nuclear Overhauser enhancement (NOE). Localized liver spectra are obtained using image selected in vivo spectroscopy (ISIS), with the resulting spectra analyzed for absolute concentration of ATP using jMRUI v4.0 software, as previously described ([14](#_ENREF_14)).

**7. Comorbidity screening**

**7.1. Neuropathy**

**7.1.1. Peripheral nerve function tests**

Peripheral nerve function tests are performed as previously described ([18](#_ENREF_18), [19](#_ENREF_19)). Quantitative sensory testing includes measurement of the vibration perception threshold (VPT) at the second metacarpal bone and medial malleolus using the method of limits (Vibrameter, Somedic, Stockholm, Sweden) and warm and cold thermal detection thresholds (TDT) at the thenar eminence and dorsum of the foot using the method of limits (TSA-II NeuroSensory Analyzer, Medoc, Ramat Yishai, Israel).

**7.1.2. Nerve fiber morphometry**

Three-millimeter skin punch biopsy specimens are taken under local anesthesia using 2% of lidocaine solution from the left lateral calf, 10 cm proximal to the lateral malleolus ([20](#_ENREF_20)). Intraepidermal nerve fiber density (IENFD) is quantified as previously described ([21](#_ENREF_21)).

Corneal confocal microscopy (CCM) is performed using a Heidelberg Retina Tomograph II (HRT II) with the Rostock Cornea Module-RCM (Heidelberg Engineering, Heidelberg, Germany) as previously described ([19](#_ENREF_19), [22](#_ENREF_22)).

**7.1.3. Autonomic function tests**

Cardiovascular autonomic nerve function is evaluated by measuring heart rate variability (HRV) during spontaneous breathing over 5 min (coefficient of R-R interval variation, spectral analysis), at deep breathing (expiration/inspiration ratio), after standing up (max/min 30:15 ratio), and in response to a Valsalva maneuver (Valsalva ratio) using VariaCardio TF5 (MIE Ltd, Leeds, UK) as previously described ([23](#_ENREF_23)). Cardiovascular autonomic neuropathy (CAN) was defined using previously defined criteria ([23](#_ENREF_23)). In addition, HRV is measured during the hyperinsulinemic-euglycemic clamp using a digital SpiderView Holter recorder (Sorin Group, Munich, Germany) ([24](#_ENREF_24)).

Spontaneous baroreflex sensitivity is assessed using the Finometer (Finapres Medical Systems, Amsterdam, The Netherlands) as previously described ([25](#_ENREF_25), [26](#_ENREF_26)).

Pupillography is used to quantify pupillary reflexes (CIP 6.00, AMTech, Dossenheim, Germany) as previously reported ([27](#_ENREF_27)). Sudomotor function is assessed by the Neuropad indicator test as previously reported ([28](#_ENREF_28)).

Laser Doppler flowmetry is performed using micro-lightguide tissue spectrophotometry (O2C) at resting conditions and during postocclusive hyperemia as previously described ([29](#_ENREF_29)).

Sexual function is assessed using the International Index of Erectile Function (IIEF) for men ([30](#_ENREF_30)) and the Kurzfragebogen für sexuelle Probleme bei Frauen (KFSP-F) for women ([31](#_ENREF_31)).

**7.2. Ophthalmology**

Central 1-field digital fundus image using either a 30° field (Canon CR 6-45 Canon Gießen, Germany) or a 200°C field (Optos P200 Optos, Bruchsal, Germany) is used to obtain digital fundus images which are rated by trained ophthalmologists. Since 2012 in addition the following program is conducted: best-corrected visual acuity is measured using Early Treatment Diabetic Retinopathy Study (ETDRS) charts. Clinical data obtained include contrast sensitivity (Mars contrast sensitivity test), slit lamp biomicroscopy and fundus examination. Corneal sensitivity of the central cornea and in all peripheral quadrants is quantified by the Luneau test. In addition to routine ophthalmic examinations, for each eye spectral-domain optical coherence tomography (SD-OCT, Spectralis® HRA+OCT, Heidelberg Engineering, Heidelberg, Germany) is performed. The high-speed resolution mode is used to collect the images. Circular B-scans (3.6-mm diameter) centered at the optic disc are generated and automatically averaged by 100 single scan to reduce speckle noise. The instrument uses 1,024 A-scan points from a 3.45 mm circle centered on the optic nerve head. The acquisition rate is 40,000 A-scans per second at a digital axial resolution of 3.9 μm. The second scan is a posterior p-pole (30°x25°, OCT volume scan), which consists of 60 scans averaged from 20 A-scans. Moreover a horizontal macula dense volume scan (30x20°) with 49 scans each consisting with 16 high speed (512 A-scans/B-scans) is obtained. The forth scan consists of 6 single scan with a deviation of 30°.

**7.3. Cardiorespiratory fitness**

Continuous measurement of oxygen uptake and carbon dioxide output during ergospirometry (Ultima CPX, Medical Graphics, Gloucester, UK; Ergometrix 900, Ergoline, Bitz, Germany) allows accurate determination of oxygen uptake at the anaerobic threshold (AT), at the respiratory compensation point (RCP) and at maximum work load (VO_2_max). The Wasserman-graphic is used to evaluate the test ([32](#_ENREF_32)).

**7.4. Vascular function**

Endothelial function is measured as flow-mediated vasodilatation (FMD) of the brachial artery ([33](#_ENREF_33)). The diameter of the brachial artery is measured 2-3 cm above the cubital fossa before and after ischemia of the forearm by a Philips Sono EnVisor C HD with 5-12 MHz transducer (Philips GmbH, Hamburg, Germany) and automatic edge-detection software (Brachial Analyzer, Medical Imaging Applications, Iowa City, Iowa, USA). The measurements are performed in the morning after an overnight fasting period, in a quiet and slightly shade room of constant temperature (23°C). Before and 90s after reactive hyperemia, induced by 5 min of distal lower arm occlusion, the diameter is assessed and FMD calculated as relative diameter gain compared with baseline. Endothelium-independent dilation is measured 4 min after sublingual application of 400 µg glycerol trinitrate.

**7.5. Patient reported measures and socio-economic status**

**7.5.1. Health-related quality of life and depression**

Health-related quality of life is assessed by the short form 36 health survey questionnaire (SF36), the World Health Organization 5 questions health survey (WHO5) ([34](#_ENREF_34), [35](#_ENREF_35)), and the World Health Organisation quality of life assessment (WHOQOL-Bref) ([36](#_ENREF_36)). Depression is assessed by the symptom checklist 14 (SCL-14) the patient health questionnaire (PHQ) ([37](#_ENREF_37)), and the Allgemeine Depressionsskala (ADS-L) ([38](#_ENREF_38)). By the problem areas in diabetes (PAID) ([39](#_ENREF_39), [40](#_ENREF_40)), diabetes-specific loads are assessed.

**7.5.2. Participation preferences, information needs, time for health-related activities**

Information needs and participation preferences are assessed by the Control Preferences Scale (CPS) and the Autonomy Preference Index (API) ([35](#_ENREF_35), [41](#_ENREF_41)) and a questionnaire developed and validated by the DDZ ([42](#_ENREF_42)). Time needed for health related activities and diabetes self management are assessed by a questionnaire developed and validated by DDZ and established instruments used in large population-based cohort studies ([42](#_ENREF_42)).

**7.5.3. Socio-economic position**

For the assessment of socioeconomic status, different measures are used. The Helmert index is used as a measure of global socioeconomic status ([43](#_ENREF_43)), and income, educational level, occupational status are used as single dimensions of the socioeconomic status.

**8. Lifestyle**

**8.1. Nutrition assessment**

The diet of the participants including alcohol intake, is assessed using food frequency questionnaires (FFQ). Two sets of FFQs (FFQ1 and FFQ2) have been used since study initiation. FFQ1 retrospectively inquired the food consumption frequencies during the last three months or four weeks, respectively. The first version of FFQ1 was limited to the qualitative assessment of the dietary intake, whereas the second version of FFQ1 additionally included a categorization of the consumed portion size (small; medium; large) ([45](#_ENREF_45)). FFQ2 is used since August 2012 within the GDS and was designed and validated within the European Prospective Investigation into Cancer and Nutrition (EPIC)-Potsdam ([46](#_ENREF_46)). It is a semi-quantitative FFQ asking for the food consumption frequencies within the last twelve months for an average portion size. FFQ2 is complemented by a questionnaire addressing carbohydrate and fat quality in more detail.

**8.2. Physical activity**

Physical activity is assessed by a standardized international questionnaire ([47](#_ENREF_47)) which has been validated in the German population ([48](#_ENREF_48)), covering occupational and leisure time physical activity and sports. Occupational physical activity and intensity of sports are classified using a standard compendium to obtain weighting factors to calculate professional, leisure time activity and sports index ([48](#_ENREF_48)).

**8.3. Smoking**

Smoking habits are assessed by a standardized questionnaire in order to obtain details on current smoking (quantitative data), former smoking (duration and time since cessation) and never smoking.

**9. Statistical analyses**

**9.1. Analysis of baseline data**

Phenotypical descriptions of the cohort at baseline are provided with standard descriptive numerical and graphical methods as, for example, measures of central tendency and variability, histograms, or boxplots. Associations between baseline variables are investigated with standard methods of correlation and regression analysis taking into account the scale (that is, continuous, binary, ordinal, or nominal) of the respective variables.

**9.2. Analysis of follow-up data**

The design of the GDS as a cohort with repeated assessment of follow-up data leads to the application of various regression models and the underlying statistical tools within such models. Due to the differing time intervals between baseline and follow-up for different participants and in order to account for censoring, regression analysis for duration data (e.g., the Cox Proportional Hazard model) will play a prominent role. If necessary, mixed or generalized estimation equation (GEE) models will be applied in order to adjust for repeated observations.

**References**

1. Nowotny B, Nowotny PJ, Strassburger K, Roden M. Precision and accuracy of blood glucose measurements using three different instruments. Diabetic medicine : a journal of the British Diabetic Association. 2012;29:260-265.

2. Greenbaum CJ, Mandrup-Poulsen T, McGee PF, et al. Mixed-meal tolerance test versus glucagon stimulation test for the assessment of beta-cell function in therapeutic trials in type 1 diabetes. Diabetes care. 2008;31:1966-1971.

3. Pacini G, Tonolo G, Sambataro M, et al. Insulin sensitivity and glucose effectiveness: minimal model analysis of regular and insulin-modified FSIGT. The American journal of physiology. 1998;274:E592-599.

4. DeFronzo RA, Tobin JD, Andres R. Glucose clamp technique: a method for quantifying insulin secretion and resistance. The American journal of physiology. 1979;237:E214-223.

5. Lehto M, Tuomi T, Mahtani MM, et al. Characterization of the MODY3 phenotype. Early-onset diabetes caused by an insulin secretion defect. The Journal of clinical investigation. 1997;99:582-591.

6. Kahl S, Nowotny B, Piepel S, et al. Estimates of insulin sensitivity from the intravenous-glucose-modified-clamp test depend on suppression of lipolysis in type 2 diabetes: a randomised controlled trial. Diabetologia. 2014;57:2094-2102.

7. Bischof MG, Bernroider E, Krssak M, et al. Hepatic glycogen metabolism in type 1 diabetes after long-term near normoglycemia. Diabetes. 2002;51:49-54.

8. Kahl S, Strassburger K, Nowotny B, et al. Comparison of liver fat indices for the diagnosis of hepatic steatosis and insulin resistance. PloS one. 2014;9:e94059.

9. Herder C, Baumert J, Zierer A, et al. Immunological and cardiometabolic risk factors in the prediction of type 2 diabetes and coronary events: MONICA/KORA Augsburg case-cohort study. PloS one. 2011;6:e19852.

10. Herder C, Bongaerts BW, Rathmann W, et al. Association of subclinical inflammation with polyneuropathy in the older population: KORA F4 study. Diabetes care. 2013;36:3663-3670.

11. Bizhang M, Ellerbrock B, Preza D, et al. Detection of nine microorganisms from the initial carious root lesions using a TaqMan-based real-time PCR. Oral diseases. 2011;17:642-652.

12. Joehanes R, Ying S, Huan T, et al. Gene expression signatures of coronary heart disease. Arteriosclerosis, thrombosis, and vascular biology. 2013;33:1418-1426.

13. Szendroedi J, Yoshimura T, Phielix E, et al. Role of diacylglycerol activation of PKCtheta in lipid-induced muscle insulin resistance in humans. Proceedings of the National Academy of Sciences of the United States of America. 2014;111:9597-9602.

14. Laufs A, Livingstone R, Nowotny B, et al. Quantitative liver 31P magnetic resonance spectroscopy at 3T on a clinical scanner. Magnetic resonance in medicine : official journal of the Society of Magnetic Resonance in Medicine/Society of Magnetic Resonance in Medicine. 2014;71:1670-1675.

15. Storlien L, Oakes ND, Kelley DE. Metabolic flexibility. The Proceedings of the Nutrition Society. 2004;63:363-368.

16. Schadewaldt P, Nowotny B, Strassburger K, Kotzka J, Roden M. Indirect calorimetry in humans: a postcalorimetric evaluation procedure for correction of metabolic monitor variability. The American journal of clinical nutrition. 2013;97:763-773.

17. Schrauwen-Hinderling VB, Kooi ME, Hesselink MK, et al. Impaired in vivo mitochondrial function but similar intramyocellular lipid content in patients with type 2 diabetes mellitus and BMI-matched control subjects. Diabetologia. 2007;50:113-120.

18. Ziegler D, Mayer P, Muhlen H, Gries FA. The natural history of somatosensory and autonomic nerve dysfunction in relation to glycaemic control during the first 5 years after diagnosis of type 1 (insulin-dependent) diabetes mellitus. Diabetologia. 1991;34:822-829.

19. Ziegler D, Papanas N, Zhivov A, et al. Early detection of nerve fiber loss by corneal confocal microscopy and skin biopsy in recently diagnosed type 2 diabetes. Diabetes. 2014;63:2454-2463.

20. Strom A, Bruggemann J, Ziegler I, et al. Pronounced reduction of cutaneous Langerhans cell density in recently diagnosed type 2 diabetes. Diabetes. 2014;63:1148-1153.

21. Lauria G, Bakkers M, Schmitz C, et al. Intraepidermal nerve fiber density at the distal leg: a worldwide normative reference study. Journal of the peripheral nervous system. 2010;15:202-207.

22. Tavakoli M, Ferdousi M, Petropoulos IN, et al. Normative Values for Corneal Nerve Morphology Assessed Using Corneal Confocal Microscopy: A Multinational Normative Data Set. Diabetes care. 2015;38:838-843.

23. Ziegler D, Laux G, Dannehl K, et al. Assessment of cardiovascular autonomic function: age-related normal ranges and reproducibility of spectral analysis, vector analysis, and standard tests of heart rate variation and blood pressure responses. Diabetic medicine : a journal of the British Diabetic Association. 1992;9:166-175.

24. Heart rate variability: standards of measurement, physiological interpretation and clinical use. Task Force of the European Society of Cardiology and the North American Society of Pacing and Electrophysiology. Circulation. 1996;93:1043-1065.

25. Ziegler D, Laude D, Akila F, Elghozi JL. Time- and frequency-domain estimation of early diabetic cardiovascular autonomic neuropathy. Clinical autonomic research : official journal of the Clinical Autonomic Research Society. 2001;11:369-376.

26. Westerhof BE, Gisolf J, Stok WJ, Wesseling KH, Karemaker JM. Time-domain cross-correlation baroreflex sensitivity: performance on the EUROBAVAR data set. Journal of hypertension. 2004;22:1371-1380.

27. Straub RH, Thies U, Jeron A, Palitzsch KD, Scholmerich J. Valid parameters for investigation of the pupillary light reflex in normal and diabetic subjects shown by factor analysis and partial correlation. Diabetologia. 1994;37:414-419.

28. Ziegler D, Papanas N, Roden M, Group GDCS. Neuropad: evaluation of three cut-off points of sudomotor dysfunction for early detection of polyneuropathy in recently diagnosed diabetes. Diabetic medicine : a journal of the British Diabetic Association. 2011;28:1412-1415.

29. Beckert S, Witte MB, Konigsrainer A, Coerper S. The impact of the Micro-Lightguide O2C for the quantification of tissue ischemia in diabetic foot ulcers. Diabetes care. 2004;27:2863-2867.

30. Rosen RC, Riley A, Wagner G, Osterloh IH, Kirkpatrick J, Mishra A. The international index of erectile function (IIEF): a multidimensional scale for assessment of erectile dysfunction. Urology. 1997;49:822-830.

31. Dieckmann R, Oelke M, Uckert S, et al. [The prevalence of sexual problems in female medical students]. Der Urologe. Ausg. A. 2004;43:955-959; quiz 959-962.

32. Kacerovsky-Bielesz G, Chmelik M, Ling C, et al. Short-term exercise training does not stimulate skeletal muscle ATP synthesis in relatives of humans with type 2 diabetes. Diabetes. 2009;58:1333-1341.

33. Corretti MC, Anderson TJ, Benjamin EJ, et al. Guidelines for the ultrasound assessment of endothelial-dependent flow-mediated vasodilation of the brachial artery: a report of the International Brachial Artery Reactivity Task Force. Journal of the American College of Cardiology. 2002;39:257-265.

34. Brazier JE, Roberts J. The estimation of a preference-based measure of health from the SF-12. Medical care. 2004;42:851-859.

35. Greiner W, Claes C, Busschbach JJ, von der Schulenburg JM. Validating the EQ-5D with time trade off for the German population. The European journal of health economics : HEPAC : health economics in prevention and care. 2005;6:124-130.

36. Brähler EM, H.; Albani, C.; Schmidt, S. . Teststatistische Prüfung und Normierung der deutschen Versionen des EUROHIS-QOL Lebensqualität-Index und des WHO-5 Wohlbefindens-Index. . Diagnostica 2007;53:83–96.

37. Bengel JW, M.; Zwingmann C. (Hg.) Diagnostische Verfahren in der Rehabilitation. Abschnitt B2 Diabtetes. Hogrefe. 2008;5 Bände.

38. Stein JL, M.; Mahnke, J.; Weyerer S.; Schomerus, G.; Riedel-Heller, SG. . Depressionsscreening am Telefon mittels der Allgemeinen Depressionsskala (ADS) Ergebnisse einer bevölkerungsrepräsentativen Erhebung Depressionsscreening am Telefon mittels der Allgemeinen Depressionsskala 2014;41:135–141.

39. Löwe BS, RL.; Zipfel, S.; Herzog, W. . PHQ-D Manual Komplettversion und Kurzform, Autorisierte deutsche Version des „Prime MD Patient Health Questionnaire (PHQ). Pfizer. 2002:1-11.

40. Gräfe KZ, S.; Herzog, W.; Löwe, B. . Screening psychischer Störungen mit dem“Gesundheitsfragebogen für Patienten (PHQ-D)“. Diagnostica 2004;50:171–181.

41. Degner LF, Russell CA. Preferences for treatment control among adults with cancer. Research in nursing & health. 1988;11:367-374.

42. Schunk M, Schweikert B, Gapp O, et al. Time trends in type 2 diabetes patients' disease management and outcomes: evidence from two KORA surveys in Germany. Experimental and clinical endocrinology & diabetes : official journal, German Society of Endocrinology [and] German Diabetes Association. 2009;117:88-94.

43. Kowall B, Rathmann W, Strassburger K, Meisinger C, Holle R, Mielck A. Socioeconomic status is not associated with type 2 diabetes incidence in an elderly population in Germany: KORA S4/F4 cohort study. Journal of epidemiology and community health. 2011;65:606-612.

44. Toeller M FA. Questionnaire to assess nutritional intake in risk groups. Diabetologia.S:309-314.

45. Noethlings U, Hoffmann K, Bergmann MM, Boeing H, European Investigation into C, Nutrition. Portion size adds limited information on variance in food intake of participants in the EPIC-Potsdam study. The Journal of nutrition. 2003;133:510-515.

46. Baecke JA, Burema J, Frijters JE. A short questionnaire for the measurement of habitual physical activity in epidemiological studies. The American journal of clinical nutrition. 1982;36:936-942.

47. Wagner P SR. Ein Fragebogen zur Erfassung der habituellen körperlichen Aktivität verschiedener Bevölkerungsgruppen. Sportwissenschaften. 2003;33:383-395.

48. Hsieh FY, Bloch DA, Larsen MD. A simple method of sample size calculation for linear and logistic regression. Statistics in medicine. 1998;17:1623-1634.
