# Supplementary material for: Cohort profile: the German Diabetes Study (GDS)
Source: Cardiovasc Diabetol. 2016 Apr 7;15:59. doi: 10.1186/s12933-016-0374-9 (PMC4823856; doi:10.1186/s12933-016-0374-9)
Supplement: Supplementary file 2 — 10.1186/s12933-016-0374-9 Risk analysis and risk mitigation measures. [file 12933_2016_374_MOESM2_ESM.docx]

**Annexure II**

**Risk analysis and risk mitigation measures**

Strength, weakness, opportunities and threat (SWOT) analysis was done before initiation of the German diabetes study (GDS) to develop risk mitigation strategies.

| **Strength** | **Weakness** |
| --- | --- |
| - High prevalence and relevance of the studied disease.  -Deep phenotyping in both type 1 and type 2 diabetes patients.  - 10 years follow up, frequent reexaminations.  - Rapid implication of technology development and novel techniques.  - Diversified funding by ministry of health, Germany, state of North Rhine-Westphalia, Germany and third party funding for individual research projects within the GDS. | - Bias towards higher educated and highly motivated patients due to time-consuming, demanding examinations.  - Hypothesis generating study design.  - Up to now no continuous control group.  - Low incidence of expected hard end-points within the follow-up period of 10 years. |
| **Opportunities** | **Threats** |
| - Combination of diverse research areas.  - International collaborations.  - National spread motivation of new participating centers all over Germany.  - Implementation of a healthy control group. | - High effort needed to reach a good respond in the long term follow-up.  - Management demands.  - Reliance on high compliance of participants. |
